# Supplementary material for: Extracorporeal Shock Wave Enhances the Cisplatin Efficacy by Improving Tissue Infiltration and Cellular Uptake in an Upper Urinary Tract Cancer Animal and Human-Derived Organoid Model
Source: Cancers (Basel). 2021 Sep 11;13(18):4558. doi: 10.3390/cancers13184558 (PMC8471724; doi:10.3390/cancers13184558)
Supplement: Supplementary file 1 [file cancers-13-04558-s001.zip › cancers-1341120-supplementary.pdf]

Supplementary Materials

Extracorporeal Shock Wave Enhances the Cisplatin Efficacy by Improving Tissue Infiltration and Cellular Uptake in an Upper Urinary Tract Cancer Animal and Human-Derived Organoid Model

Hao-Lun Luo, Hui-Ying Liu, Yin-Lun Chang, Yu-Li Su, Chun-Chieh Huang, Xin-Jie Lin and Yao-Chi Chuang

Table S1. Summary of patient-derived UTUC organoid corresponding clinical data.

Sample	Age	Gender	Pathological t Stage	Histological Grade	Lymphovascular Invasion	CIS	Tumour Necrosis
KCGMH-01	73	male	Ta	high	negative	negative	positive
KCGMH-02	87	female	T3	high	positive	positive	positive
KCGMH-03	80	male	T2	high	negative	negative	negative
KCGMH-04	58	female	T1	high	negative	positive	positive
KCGMH-05	50	male	T3	high	positive	positive	negative
KCGMH-06	72	male	T3	high	positive	negative	positive

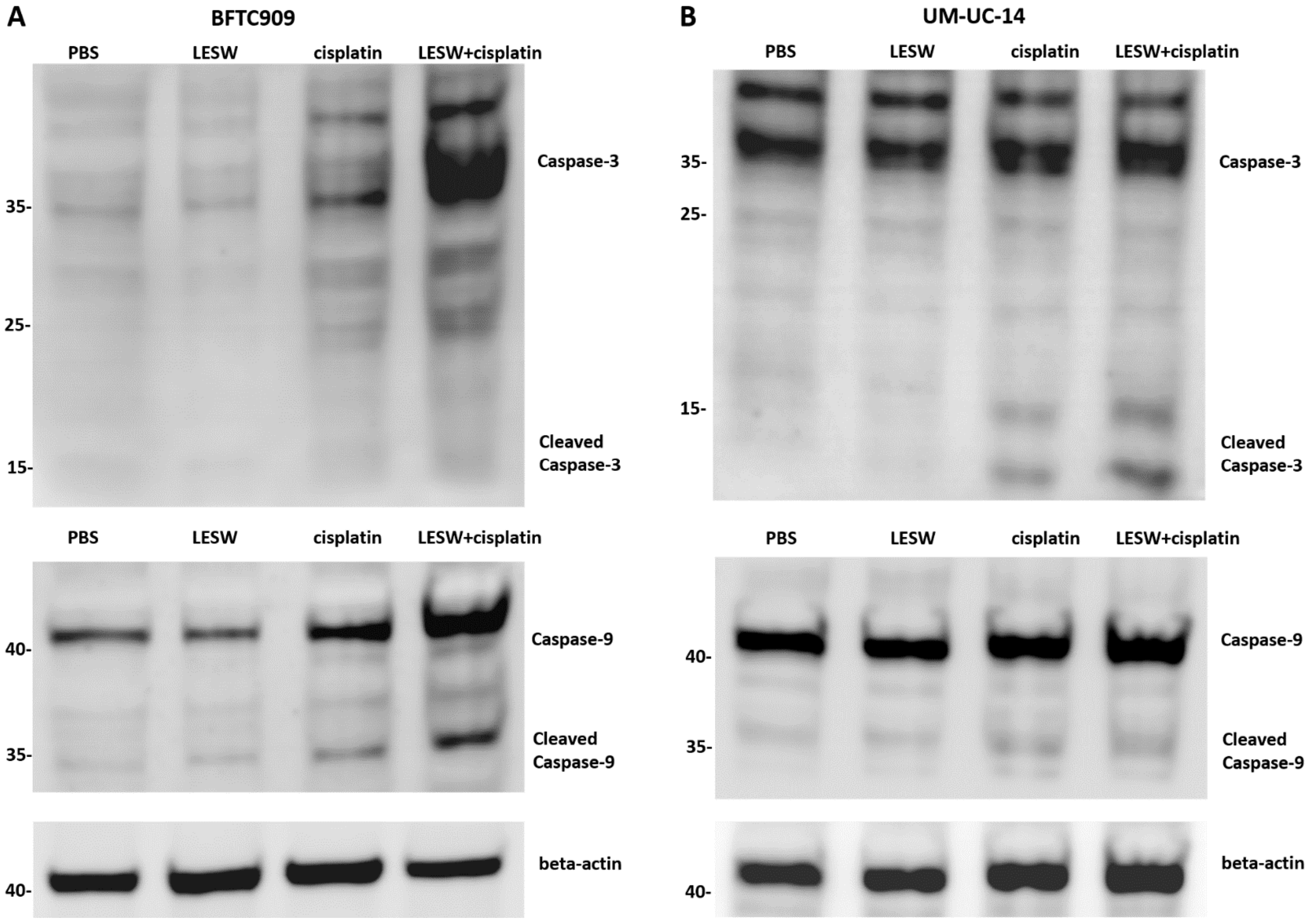

Figure S1. The combination of cisplatin and shock waves enhanced the activation of caspase-3 and caspase-9 apoptosis signaling. Western blot analysis of procaspase-3 and cleaved caspase 3 (top), procaspase-9 and cleaved caspase 9 (middle), and beta-actin (bottom) from BFTC909 (**A**) and UM-UC-14 (**B**) cells after incubation with PBS (control), LESW (200 shock

wave pulses at 0.12 mJ/mm²), cisplatin (3 μM) and LESW+cisplatin (3 μM cisplatin combined with 200 shock wave pulses at 0.12 mJ/mm²) for 48 h.

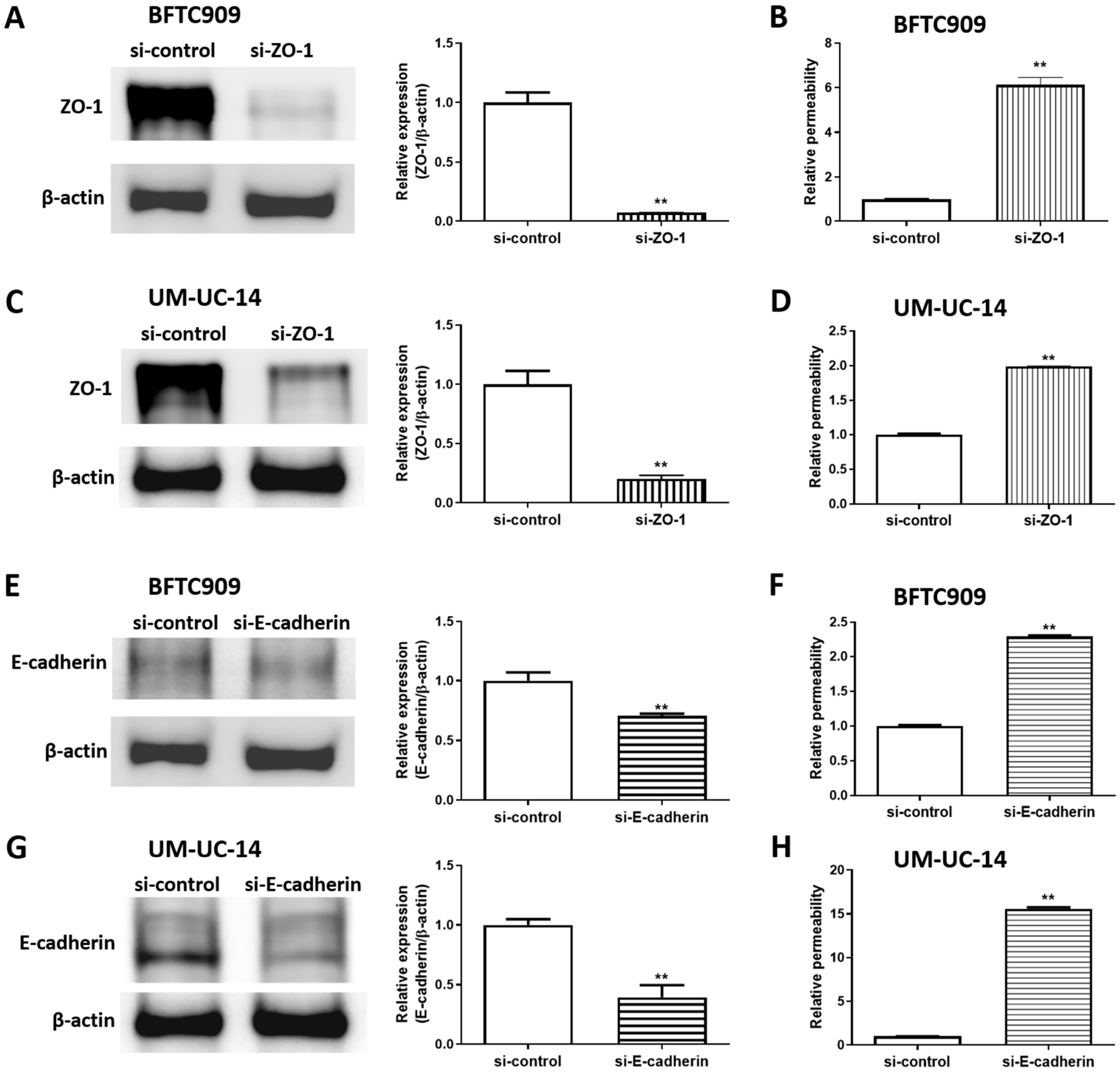

Figure S2. RNAi-mediated down-regulation of ZO-1 or E-cadherin increases the permeability in BFTC909 and UM-UC-14 cells using in vitro permeability assay. BFTC909 and UM-UC-14 cells were transfected with pools of siRNA for ZO-1 or E-cadherin in the Human siGENOME® SMARTpool® siRNA. The lysates from BFTC909 (**A**) and UM-UC-14 (**C**) cells transfected with siRNA for ZO-1 were analysed by Western blotting. Relative permeability of BFTC909 (**B**) and UM-UC-14 (**D**) cells after knockdown of ZO-1 was detected by in vitro permeability assay. BFTC909 (**E**) and UM-UC-14 (**G**) cells transfected with siRNA for E-cadherin were then subjected to Western blots with indicated antibodies. Relative permeability of BFTC909 (**F**) and UM-UC-14 (**H**) cells after knockdown of E-cadherin was detected by in vitro permeability assay. Error bars represent mean ± S.E.M., the p values were calculated with Student's t-test, ** $p < 0.01$ versus si-control group.

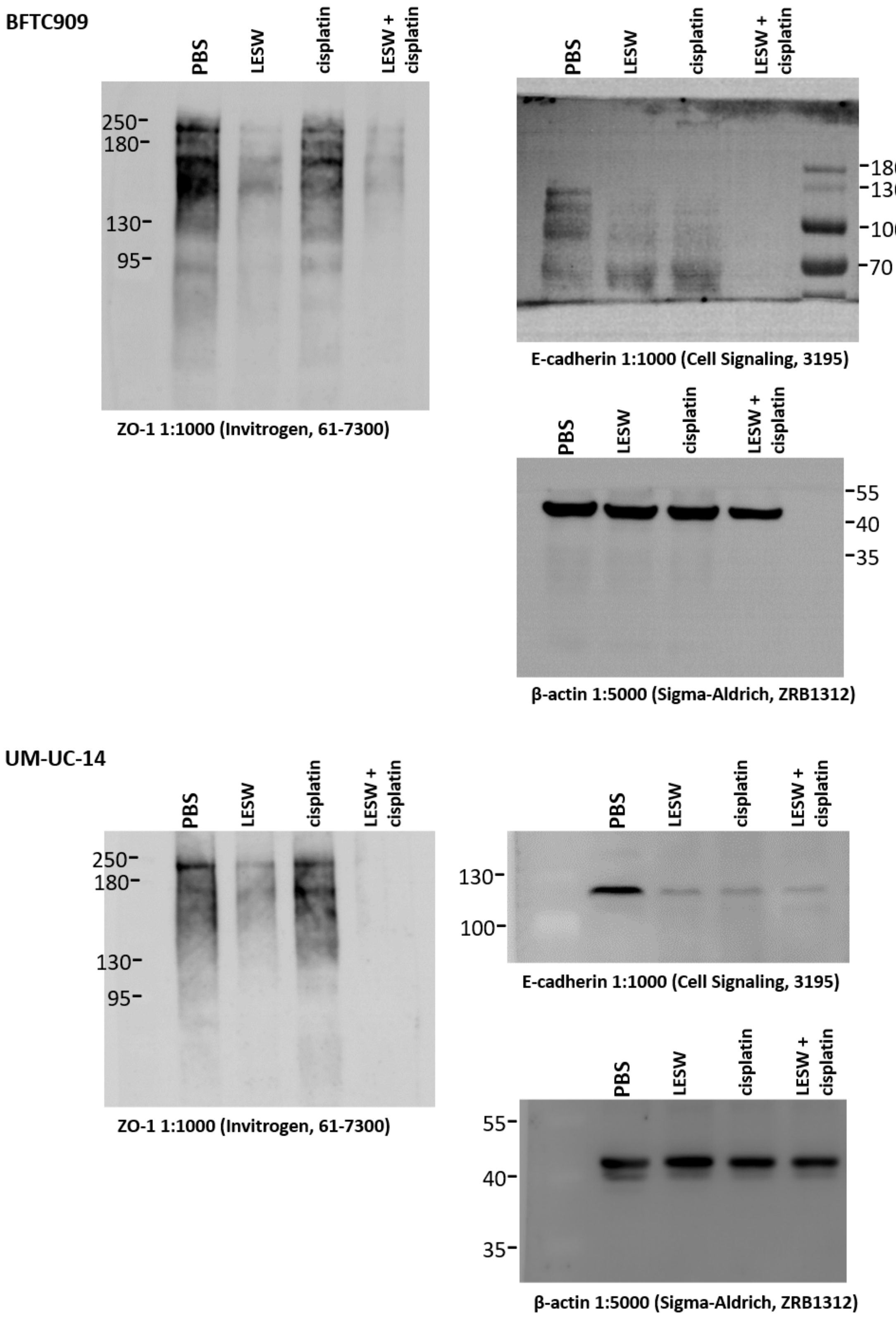

Figure S3. Full unedited gels for Figure 4C.

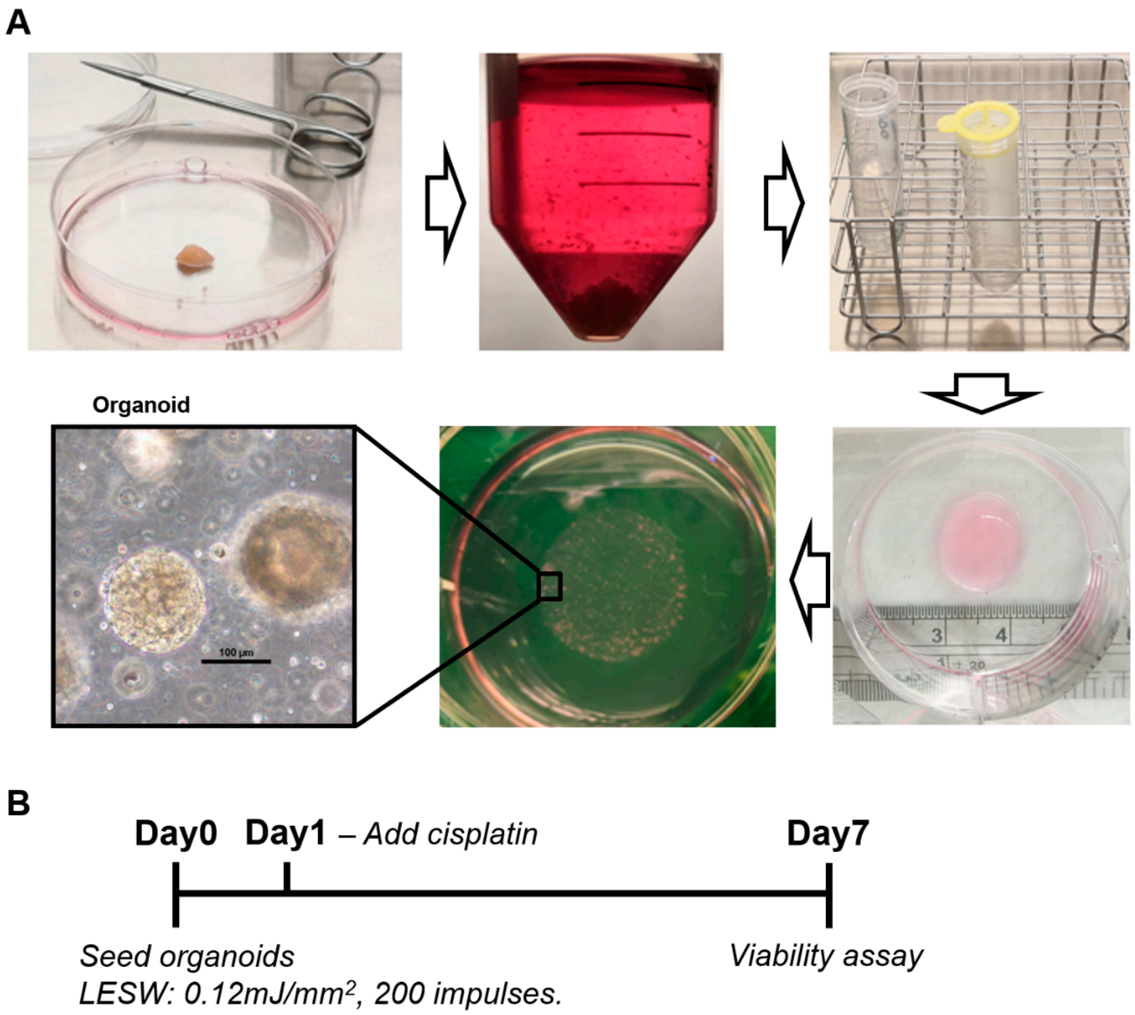

Figure S4. Detail information about UTUC PDO culture and drug assay with shock wave treatment. (A) Tumour tissues were minced with scissors and incubated in 10 mL of the organoid culture medium supplemented with 1 mL collagenase/hyaluronidase at 37 °C for 15 min. Dissociated tissues were spun down at 300 g for 5 min, resuspended in 10 mL of PBS, and spun down again. The tissues were resuspended in 5 mL of TrypLE Express and incubated at room temperature for 3 min. Dissociated tissues were spun down at 300 g for 5 min, resuspended in 10 mL of HBSS supplemented with 5% charcoal-stripped FBS, 10 mM Y-27632, and 100 mg/mL Primocin, and passed through a 100 μm cell strainer. Dissociated cells (1×10^6 cells/well) were spun down at 300 g for 5 min, resuspended in 60% Matrigel/organoid culture medium, plated in a 250 μL drop in the middle of one well of a pre-coated 6-well plate with 60% Matrigel, and solidified at 37 °C for 30 min. After solid drops formed, 1.5 mL of the organoid culture media was added to the well. (B) Patient-derived organoids were collected after passaging and passed through a 100 μm cell strainer to eliminate large organoids. Subsequently, organoids were resuspended in 2% Matrigel/organoid culture medium in 15-mL polypropylene tubes and spun down at 300 g for 5 min. The bottom of the tubes was covered with ultrasound transmission gel. The EvoTron™ shock wave applicator was gently placed directly on the bottom of the tube. The shock wave frequency was 4 pulses per second. Organoids were exposed to 200 shock wave pulses at 0.12 mJ/mm^2 and dispensed into ultralow-attachment 96-well plates in triplicate. At 24 h after plating, organoids were exposed to 0.16, 0.8, or 4 μM cisplatin for 6 days of drug incubation, and cell viability was detected by CellTiter-Glo 3D assay.
